# Nitric oxide regulates cytochrome P450 2D6 and 3A4 activity *via* concentration-dependent modulation of heme loading

**DOI:** 10.1016/j.jbc.2025.110772

**Published:** 2025-09-27

**Authors:** Priya Das Sinha, Sidra Islam, Pranjal Biswas, Dennis J. Stuehr

**Affiliations:** Department of Inflammation and Immunity, Cleveland Clinic Lerner College of Medicine of Case Western Reserve University School of Medicine, Cleveland, Ohio, USA

**Keywords:** heme protein, mitochondria, heme trafficking, Hsp90, GAPDH

## Abstract

Cytochrome P450 enzymes (CYPs) play diverse roles in human health and disease, and although their activities depend on their heme contents, the cellular mechanisms governing CYP heme levels are unclear. Because CYP activities are influenced by biological nitric oxide (NO), we investigated how a range of NO exposures would impact the heme levels and activities of CYP2D6 and 3A4 expressed in Chinese hamster ovary cells and in the human liver cell line HepG2. Following expression, both CYPs were present as a 60:40 mix of heme-free and heme-bound forms. A low range of NO concentrations (approximately 1–10 nM) generated in cultures by a chemical NO donor or by added activated macrophages caused cells to allocate heme into their heme-free CYP3A4 and 2D6 populations such that the levels of heme-replete and active CYPs increased by twofold to threefold. NO concentrations above this range (approximately 25–100 nM) gradually lost the positive effect and at the higher level caused heme loss from the CYPs and corresponding losses in activity. The positive or negative effects of NO began within the first 2 h of exposure and were completed within 6 h. The NO-driven increase in CYP heme content relied on a GAPDH–heme complex forming and chaperone heat shock protein 90 activity in the cells. Thus, NO can upregulate or downregulate cellular CYP3A4 and 2D6 activities by exerting a concentration-dependent change in their heme contents. These findings may help explain how NO generation in disease or inflammation can change CYP activities and impact drug pharmacokinetics and the generation of immune-active metabolites.

Cytochrome P450 enzymes (CYPs) are a superfamily of membrane-associated iron-protoporphyrin IX (heme) proteins that play a pivotal role in the metabolism of various drugs, xenobiotics, and a wide variety of both endogenous and exogenous substances (1, 2). Mammals simultaneously express various CYPs across multiple tissues, including the liver, kidney, lungs, brain, and adrenal glands, and many are associated with specific physiological functions (3, 4, 5). Approximately 90% of drug metabolism is attributed to six CYP isozymes: CYP1A2, CYP2C9, CYP2C19, CYP2D6, CYP2E1, and CYP3A4 (6). Of these, CYP3A4 and 2D6 are signature members that are mainly expressed in the liver and have been thoroughly investigated. In addition, exposure to drugs such as steroids can further enhance their expression (6, 7). CYP2D6 and 3A4 predominantly localize to the endoplasmic reticulum (8) anchored by an N-terminal transmembrane alpha-helix (8, 9, 10, 11). The extensive substrate promiscuity and considerable expression levels of CYP3A4 and 2D6 render them vital targets for drug metabolism and pharmacogenomics (12).

Because the CYP heme cofactor binds and activates dioxygen to create the reactive species involved in substrate oxidations, CYP activities are directly related to their heme contents (13, 14). In our recent study, we found that a considerable proportion of CYP3A4 and 2D6 (approximately 50–70%) existed in heme-free form after they were expressed in mammalian cells, and their maturation to heme-containing functional forms depended on the heme-trafficking ability of GAPDH and on the cell chaperone heat shock protein 90 (Hsp90) to enable their heme insertions (15). Studies by others have established that CYP activities are altered during inflammation (16, 17), which also is associated with enhanced hepatic nitric oxide (NO) production through it activating the expression of inducible nitric oxide synthase (iNOS) in liver cells (17, 18, 19, 20). NO can either exacerbate or reduce inflammation and can directly inhibit CYP enzymes in inflammatory situations by binding to the heme iron or by causing reversible or irreversible effects whose mechanisms are still unclear. The NO impacts on CYP activities can in turn influence the course of inflammation and alter drug metabolism (21, 22, 23).

We had previously found that relatively high NO exposures prevented mammalian cells from inserting heme into multiple heme proteins, including CYP2D6 and 3A4 (24, 25). More recently, we found that very low NO exposure could have an opposite effect, as indicated by it triggering mammalian cells to allocate heme into the heme-free forms of indoleamine dioxygenase (indoleamine-2,3-dioxygenase [IDO1]) and Trp dioxygenase (26). Building on this, we now investigated how a wide range of NO concentrations may impact cell heme allocation into or out of CYP3A4 and CYP2D6 when they are expressed in mammalian cells. We achieved graded levels of NO exposure by employing a well-characterized chemical NO donor or by adding different numbers of macrophage cells that were expressing iNOS and generating NO. We probed the mechanism of the NO effect on CYP heme levels by determining the concentration dependence, kinetics, and reliance on GAPDH and Hsp90. Our findings reveal that NO exposure can upregulate or downregulate CYP2D6 and 3A4 activities in cells through it having a dynamic and concentration-dependent impact on cell heme allocation that causes these CYPs to gain or lose their heme. Our findings may help explain how different levels of NO that are generated in health and disease can cause changes in CYP activities that impact their biological functions and pharmacologic profiles.

## Results

### NO exerts a concentration-dependent hormetic effect on CYP2D6 and 3A4 activities and heme contents in cells

We first examined how a range of NO exposures would impact the activities and heme contents of CYP2D6 and 3A4. Each FLAG- and MYC-tagged CYP protein was transiently expressed in glycine auxotroph Chinese hamster ovary cells (GlyA-CHO) in ^14^C-glycine (^14^C-Gly)-containing medium, which allowed the cells to generate ^14^C-heme during the CYP expression period. After 30 h of transfection, the cells were treated with cycloheximide (Chx) at a nontoxic concentration (Fig. S1) to prevent further protein expression and then cultured with different concentrations of the slow-release NO donor 2,2′-(hydroxynitrosohydrazino)*bis*-ethanolamine (NOC18) for 6 h, which releases 2 NO per mole and whose measured half-life under our particular cell culture conditions is 16 h (26). After the NOC18 incubation, the cells were harvested and supernatants were prepared. CYP activities in supernatants were obtained by measuring the rate of product formation *versus* time, whereas the relative CYP ^14^C-heme contents were determined in the same samples by immunoprecipitation (IP) with anti-MYC antibody and ^14^C scintillation counting. Controls included cells not transfected to express the CYPs and transfected cells whose heme biosynthesis had been intentionally blocked by inclusion of succinyl acetone (SA) throughout the transfection and experimental periods (25, 27). This enabled us to measure the ^14^C counts in the IPs that were due to the ^14^C-Gly contents of the CYP apoproteins. These counts ranged from 160 to 352 across the different experiments and were subtracted in each case from the total ^14^C counts obtained in the corresponding IPs to obtain the ^14^C-heme-specific counts reported in the figures.

**Figure 1:**
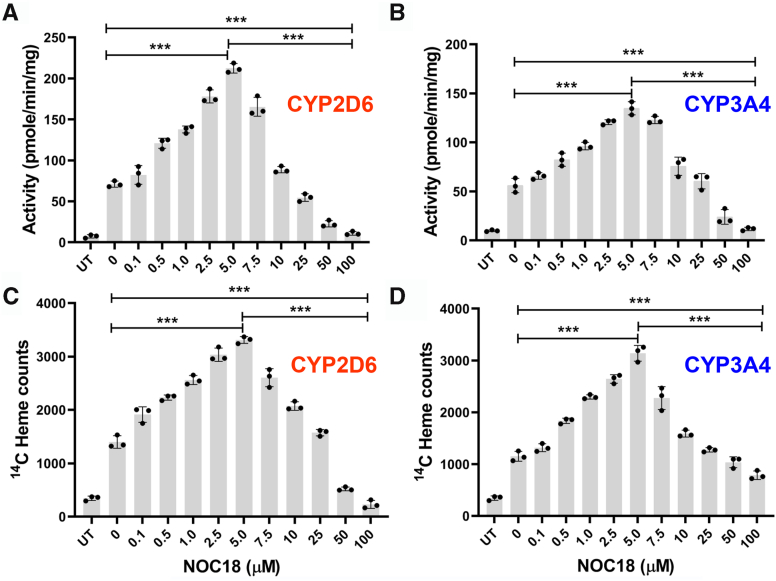
**NO governs the activities of CYP2D6 and 3A4 in cells by modulating their heme content in a concentration-dependent bimodal fashion.** GlyA-CHO cells underwent transfection to express FLAG- and MYC-tagged CYP3A4 or CYP2D6 in media enriched with ^14^C Gly for 48 h, after which protein expression was ceased by Chx addition, and NOC18 was added at the designated concentrations. The cultures were maintained for 6 h before harvesting, followed by supernatant CYP activity assay and MYC Ab pulldown of the CYP to determine heme contents. *A* and *B,* CYP2D6 and CYP3A4 activities. *C* and *D,* CYP2D6 and CYP3A4 ^14^C heme counts. Data are the mean ± SD; n = 3 independent experiments. ∗∗∗*p* < 0.001, one-way ANOVA. Ab, antibody; Chx, cycloheximide; CYP, cytochrome P450; ^14^C-Gly, ^14^C-glycine; GlyA-CHO, glycine auxotroph Chinese hamster ovary cell; NO, nitric oxide; NOC18, 2,2′-(hydroxynitrosohydrazino)bis-ethanolamine; UT, untransfected cell samples.

Figure 1, *A* and *B* shows that GlyA-CHO cells that had been cultured for 6 h with the lower range of NOC18 concentrations (0.1 through 5 μM, giving estimated steady-state NO concentrations of 0.1–5 nM) (28) had increasingly higher CYP2D6 and 3A4 activities such that at 5 μM NOC18 they were threefold greater than the activities of the CYP-transfected cells that did not receive NOC18. At NOC18 concentrations above 5 μM, the positive effect steadily diminished and was completely lost in cells that received 25 μM NOC18. In cells that received 50 or 100 μM NOC18, the CYP activities fell below those of CYP-transfected cells that had received no NOC18 and approached or matched the background activities of the untransfected cell supernatants. Western blot analyses showed that CYP protein expression levels remained uniform across the range of NOC18 exposures (Fig. S2). Essentially identical results regarding CYP3A4 activity and expression were observed in replica experiments using GlyA-CHO or HepG2 (hepatocellular carcinoma G2 cell line) cell cultures that did not receive Chx (Fig. S4).

**Figure 2:**
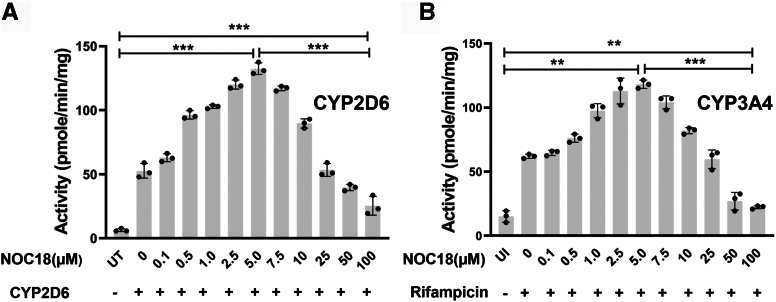
**NO governs the activities of CYP2D6 and 3A4within HepG2 cells.** HepG2 cells underwent transfection to express FLAG- and MYC-tagged CYP2D6 (*A*) or were induced by rifampicin to express CYP3A4 (*B*) in media enriched with ^14^C Gly, after which protein expression was ceased by Chx addition and NOC18 was added to give the designated concentrations. After 6 h, cells were harvested, and the supernatants were subjected to a CYP activity assay. Data are the mean ± SD; n = 3 independent experiments. ∗∗∗*p* < 0.001, ∗∗*p* < 0.01, one-way ANOVA. Chx, cycloheximide; CYP, cytochrome P450; HepG2, hepatocellular carcinoma G2 cell line; NO, nitric oxide; NOC18, 2,2′-(hydroxynitrosohydrazino)bis-ethanolamine; UT, untransfected cell samples; UI, uninduced cell samples.

Figure 1C and D shows the impact of the NOC18 treatments on the level of ^14^C-heme in the IP samples for CYP2D6 and 3A4. The CYP ^14^C-heme contents steadily rose in the cells given 0.1 to 5 μM NOC18 to reach between a twofold and threefold increase in their ^14^C-heme and then steadily fell in cells exposed to the higher NOC18 concentrations such that the 10 or 25 μM NOC18 exposures had no effect on CYP ^14^C-heme levels, and the 50 and 100 μM NOC18 exposures lowered the CYP ^14^C heme levels to those seen for the supernatant IPs of the untransfected cells, indicating a loss of their existing heme. We observed nearly identical NOC18 concentration effects on CYP3A4 or CYP2D6 activities when their expression was induced in the human liver cell line HepG2 by transfection or by rifampicin treatment, respectively (Figs. 2, *A* and *B*; S3). Thus, in all cases, the NOC18 exposures displayed a highly similar concentration–response profile in causing cells to change the catalytic activities and heme contents of their CYP2D6 and 3A4. Remarkably, both parameters displayed a hormetic response toward the NOC18 concentration such that at low concentrations it had an increasingly positive effect until a threshold was reached, after which increasing the NOC18 concentration caused a gradually diminishing positive and ultimately a negative effect.

### NO released by immune-stimulated cells alters the activity and heme content of CYP3A4 expressed in adjacent cells in coculture

**Figure 3:**
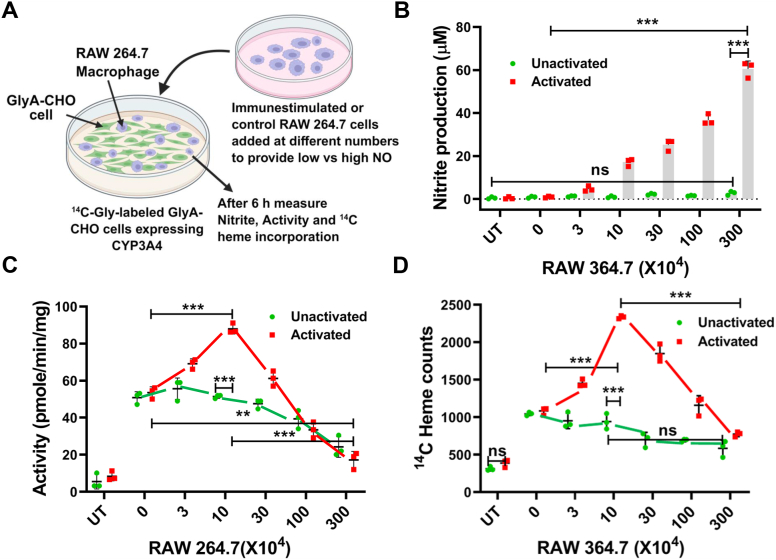
**NO generated by immune-stimulated macrophage cells modulates the heme level and activity of CYP3A4 expressed in cocultured GlyA-CHO cells.***A,* outline of the experiment; RAW264.7 cells that had been activated or not for NO synthesis by culture with bacterial LPS were added in varying numbers to monolayers of GlyA-CHO cells expressing CYP3A4 incorporated with ^14^C heme by previous culture with ^14^C-Gly. Following 6 h of coulture, the nitrite concentration in the culture fluid was quantified (*B*) along with the CYP activity of the supernatant (*C*) and the ^14^C heme counts in MYC Ab pulldowns of CYP3A4 from the supernatants (*D*). Data are the mean ± SD of three experiments. ∗∗∗*p* < 0.001, ∗∗*p* < 0.01, one-way ANOVA. Ab, antibody; ^14^C-Gly, ^14^C-glycine; CYP, cytochrome P450; GlyA-CHO, glycine auxotroph Chinese hamster ovary cell; NO, nitric oxide; UT, untransfected cell samples.

To test if NO generated by cells would have a similar effect as the NOC18, we performed a coculture experiment (Fig. 3*A*) in which RAW264.7 macrophage cells that either had or had not been given *Escherichia coli* lipopolysaccharide (LPS) (29) and interferon-γ (IFN-γ) to induce expression of iNOS and consequent NO production were added in different quantities to culture plates containing monolayers of GlyA-CHO cells expressing CYP3A4 that contained its basal level of ^14^C-heme because of the cells having been cultured with ^14^C-Gly during the transfection period. The plates received either none or five different quantities of either the control or activated RAW264.7 cells and then were further incubated as a coculture for 6 h prior to cell harvest. Figure 3*B* shows that the NO oxidation product nitrite had accumulated in the culture fluids after 6 h to levels that directly correlated with the number of activated RAW264.7 cells that had been added. Figure 3, *C* and *D* compares the supernatant CYP3A4 activities and the ^14^C-heme contents of the CYP3A4 IP samples, respectively. The CYP3A4 activities and ^14^C-heme contents for the GlyA-CHO cells that had been cocultured with different numbers of unactivated RAW264.7 cells remained similar and showed some loss at the higher RAW264.7 cell additions. This finding is consistent with our reporting the activities and counts on an equal total cell supernatant protein basis and with the supernatants containing proportionally less and less CYP protein from the GlyA-CHO cells as the numbers of added RAW264.7 cells increased. In the GlyA-CHO cocultures that had received activated RAW264.7 cells, the CYP3A4 activities and ^14^C-heme contents gradually increased until 10 × 10^4^ activated macrophage cells had been added and then gradually decreased and fell below the levels seen for the control transfected cells as the numbers of added activated cells further increased (Fig. 3, *C* and *D*). The losses in activity and CYP3A4 ^14^C-heme content in the cultures receiving higher numbers of the activated RAW264.7 cells could only partly be ascribed to the decline in the percentage of GlyA-CHO-derived protein in the cell supernatants as noted previously. Expression levels of CYP3A4 were similar across all cultures and conditions (Fig. S5, *A* and *B*). The effects seen in the coculture system with graded addition of activated macrophage cells mirrored what we observed for cells that underwent 6 h exposure to the different NOC18 concentrations as reported in Figure 1. Together, this indicates that the NO released by the activated RAW264.7 cells or by NOC18 was similar in causing a concentration-dependent hormetic change in the activity and heme content of CYP3A4 that was expressed in neighboring GlyA-CHO cells in the coculture system.

### Time course of the NO-mediated alterations in CYP activities and heme contents

**Figure 4:**
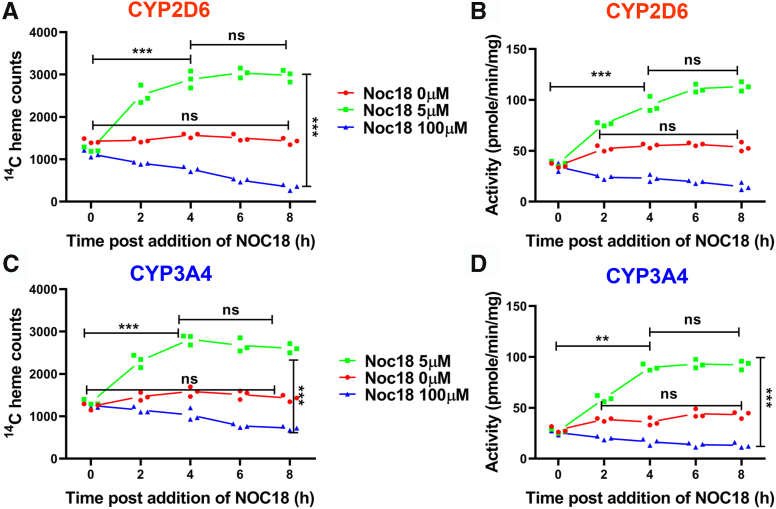
**Kinetics of NO-induced alterations in CYP450 activities and heme contents.** GlyA-CHO cells that were transfected to express CYP2D6 or 3A4 were incubated with ^14^C-Gly to generate ^14^C-labeled heme. Protein synthesis was then blocked with Chx, and NOC18 was added at concentrations of 0 (*red*), 5 (green), and 100 (*blue*) μM, followed by harvesting and analysis of the cell supernatants at designated time points. *A* and *C,*^14^C-heme counts in CYP2D6 and CYP3A4 MYC Ab pull-downs. *B* and *D,* CYP2D6 and CYP3A4 activities. Data are the mean ± SD; n = 3 experiments. ∗∗∗*p* < 0.001, ∗∗*p* < 0.01, ns = not significant, one-way ANOVA. Ab, antibody; ^14^C-Gly, ^14^C-glycine; Chx, cycloheximide; CYP450, cytochrome P450; GlyA-CHO, glycine auxotroph Chinese hamster ovary cell; NO, nitric oxide; NOC18, 2,2′-(hydroxynitrosohydrazino)bis-ethanolamine; UT, untransfected cell samples.

To investigate the kinetics of the positive and negative NO-driven changes, we expressed CYP2D6 or 3A4 in ^14^C-Gly-treated GlyA-CHO cells and then exposed them to media alone or to media containing a lower (5 μM) or higher (100 μM) concentration of NOC18 and then determined the ^14^C heme contents and activities of the CYPs *versus* time over the next 8 h. Figure 4, *A* and *C* compares the ^14^C-heme levels in CYP2D6 and 3A4 from IPs of supernatants that were made from cells harvested at each indicated time point under the three different culture conditions. In the control culture condition, both CYPs maintained relatively constant levels of ^14^C-heme throughout the time course. In cells given 5 μM NOC18, a 75% increase in the CYP ^14^C-heme contents occurred within the first 2 h of the NOC18 exposure and then continued to increase until 4-6 h, after which their ^14^C-heme contents remained at constant levels that were about two times greater than their initial levels at time = 0. In contrast, in cells given 100 μM NOC18, there was no gain in the ^14^C-heme contents of either CYP at any time point, and instead their ^14^C-heme levels steadily decreased by about 50% to 75% at 8 h relative to their time = 0 samples. Identical kinetic patterns held for cells given 5 or 100 μM NOC18 regarding a gain or loss in their supernatant CYP activities (Fig. 4, *B* and *D*). Western analyses indicated that none of the changes in heme content or activity could be attributed to changes in CYP protein expression levels, which remained similar across the time points (Figs. S6 and S7). Thus, the low NO exposure quickly caused the cells to begin steadily incorporating heme into their heme-free CYP populations, whereas the higher NO exposure stimulated no heme incorporation and instead caused a gradual heme loss from either CYP.

### NO facilitates cell heme allocation to CYP3A4 through a GAPDH-dependent mechanism

During their normal maturation process, cell heme delivery to apo-CYP3A4 or 2D6 requires the participation of a GAPDH–heme complex (27). We therefore investigated if the NO-driven heme allocation to CYP3A4 also involves GAPDH by employing our established siRNA knockdown and rescue strategy (15, 27). The GlyA-CHO cells first underwent targeted siRNA knockdown of GAPDH expression or treatment with scrambled siRNA, then were given ^14^C-Gly and transiently transfected to express CYP3A4 either alone or in combination with siRNA-resistant forms of wildtype hemagglutinin (HA)-GAPDH or the heme-binding defective HA-GAPDH-H53A variant. Cells were then given 0 or 5 μM NOC18, and the CYP3A4 ^14^C-heme contents and activities were determined after an additional 6 h of culture.

**Figure 5:**
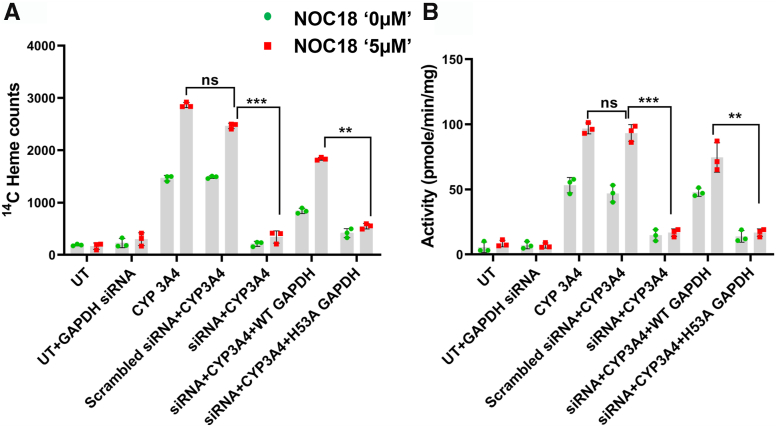
**NO stimulates cell heme insertion into CYP3A4 by a GAPDH-dependent mechanism.** GlyA-CHO cells that had been subjected to siRNA knockdown of GAPDH expression or treated with scrambled siRNA were administered ^14^C-Gly and subsequently transfected to express CYP3A4 either alone or in conjunction with siRNA-resistant versions of wildtype HA-GAPDH or the heme-binding defective HA-GAPDH-H53A variant. Cells were subsequently treated with 0 or 5 μM NOC18, and after 6 h, the cell supernatants were prepared, and their CYP3A4 ^14^C-heme levels (*A*) and the activities (*B*) were assessed. Data are the mean ± SD; n = 3 experiments. ∗∗∗*p* < 0.001, ∗∗*p* < 0.01, ns, not significant, one-way ANOVA. ^14^C-Gly, ^14^C-glycine; CYP, cytochrome P450; GlyA-CHO, glycine auxotroph Chinese hamster ovary cell; HA, hemagglutinin; NO, nitric oxide; NOC18, 2,2′-(hydroxynitrosohydrazino)bis-ethanolamine; UT, untransfected cell samples.

The targeted knockdown of GAPDH for 48 h lowered its cell expression level by approximately 90% relative to controls (Fig. S8*B*), and the expression of either HA-GAPDH protein in the knockdown cells restored their GAPDH expression level to a normal value, all without impacting the cell CYP3A4 expression levels (Fig. S8*A*). Figure 5, *A* and B shows the CYP3A4 ^14^C-heme contents and activities in the IPs and supernatants, respectively, for cells that had undergone the various described treatments. In cells that received the scrambled siRNA treatment, the CYP3A4 had normal ^14^C heme content and activity and increased both parameters in response to 5 μM NOC18 as normal. In contrast, in the cells that underwent siRNA GAPDH knockdown, the CYP3A4 had very little ^14^C heme content or activity, and these parameters were not increased significantly by the 5 μM NOC18 exposure. Expression of the wildtype HA-GAPDH in the knockdown cells restored the CYP3A4 ^14^C-heme content and activity to 60% and 90% of the levels seen for the positive control culture, respectively, and restored the ability of 5 μM NOC18 to cause an increase in these two parameters, whereas expression of the GAPDH heme-binding mutant (HA-tagged H53A GAPDH) in the knockdown cells did not. We conclude that the ability of 5 μM NOC18 to increase the ^14^C-heme content and activity of CYP3A4 in the cells depended on the expression level of GAPDH and specifically on its ability to bind intracellular heme.

### NO-stimulated heme insertion into CYP3A4 is dependent on chaperone Hsp90 activity

**Figure 6:**
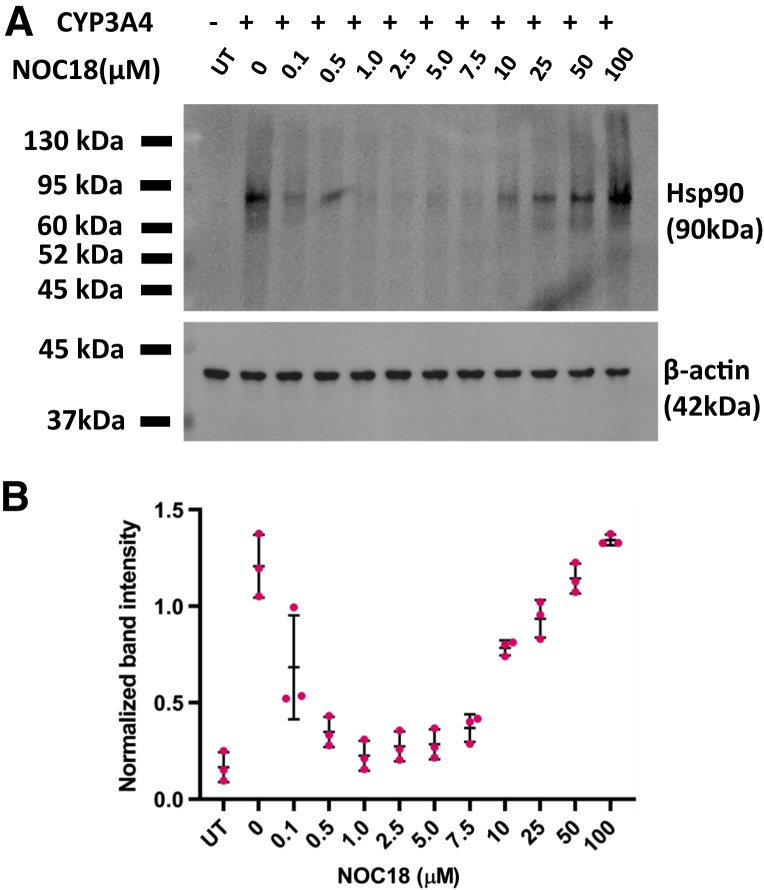
**Effect of NOC18 exposures on Hsp90 association with CYP3A4.** GlyA-CHO cells that had been transfected to express FLAG- and MYC-tagged CYP3A4 in media enriched with ^14^C-Gly were given Chx and given NOC18 at the designated concentrations, cultured for 6 h, harvested, and the cell supernatants underwent MYC Ab pulldown and Western analysis to compare bound Hsp90 levels. *A,* representative Western blot indicating the relative levels of Hsp90 associated with CYP3A4. *B,* corresponding normalized band intensities. Data are the mean ± SD of three independent experiments, ∗∗*p* < 0.01, one-way ANOVA. Ab, antibody; ^14^C-Gly, ^14^C-glycine; Chx, cycloheximide; CYP, cytochrome P450; GlyA-CHO, glycine auxotroph Chinese hamster ovary cell; Hsp90, heat shock protein 90; NOC18, 2,2′-(hydroxynitrosohydrazino)bis-ethanolamine; UT, untransfected cell samples.

Given that Hsp90 helps to drive heme insertion into CYP3A4 and 2D6 during their normal maturation process in cells (15), we investigated if Hsp90 was also involved in the NO-driven heme allocation into CYP3A4. Because Hsp90 is found associated with apo-CYP3A4 in cells (15), we tested how the level of Hsp90–CYP3A4 association would be influenced by incubating the cells for 6 h with our range of NOC18 concentrations. Results from IP and Western analysis are shown in Figure 6*A* and *B*. The CYP3A4 association with Hsp90 was relatively strong in the GlyA-CHO cells that received no NOC18. In the cell cultures that received increasing NOC18 concentrations, there was first a gradual loss and then a regain in the Hsp90–CYP3A4 association. The change in Hsp90 association level was the inverse of how the same NOC18 concentrations affected the CYP3A4 activity and heme levels (Fig. 1). This pattern is consistent with Hsp90 associating with inactive forms of CYP3A4 in cells and suggests that the level of its Hsp90 association is linked to NOC18-driven gains or losses in its heme content.

**Figure 7:**
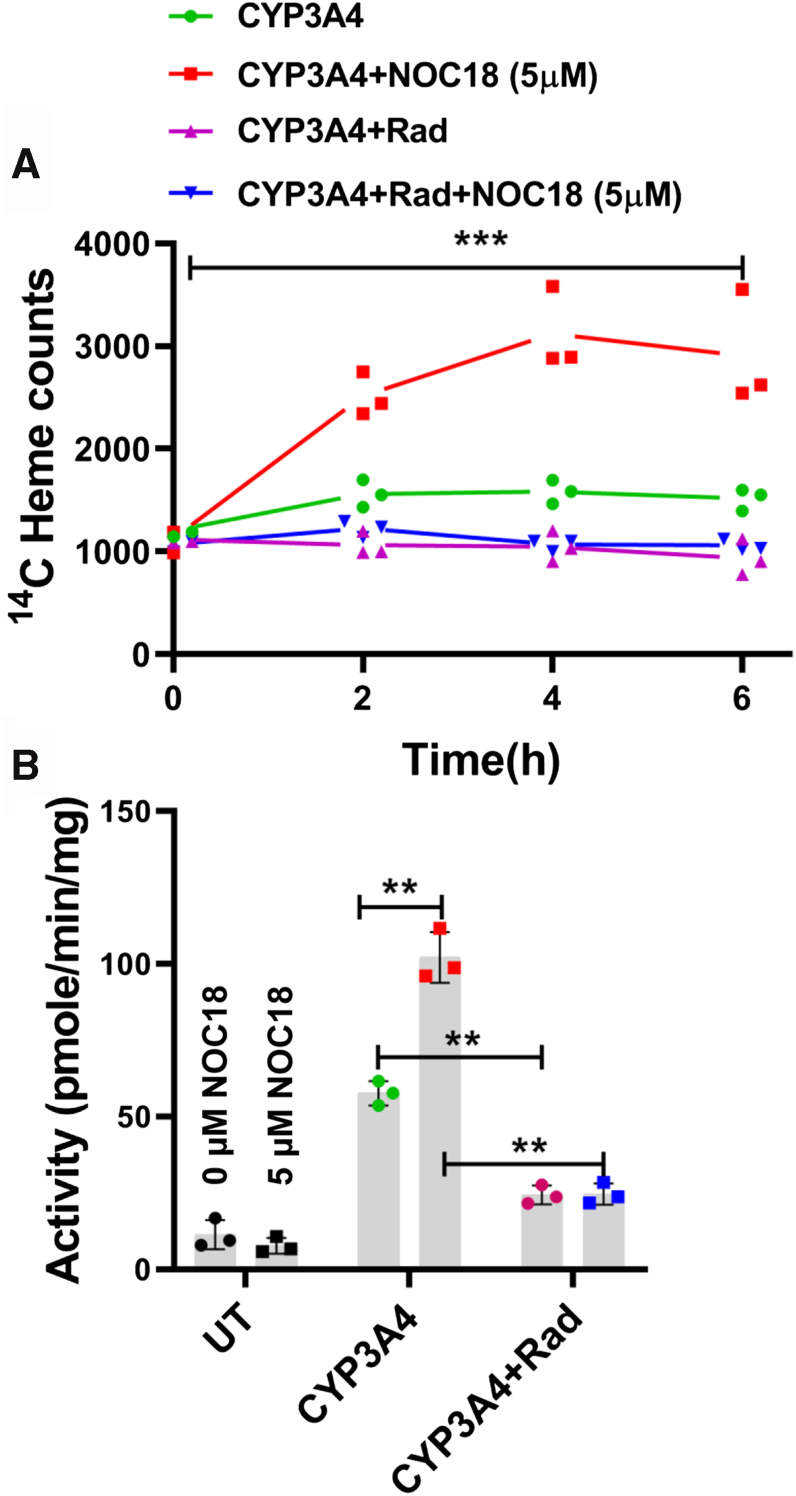
**NO-driven heme insertion in CYP3A4 requires cell Hsp90 activity.** GlyA-CHO cells underwent transfection to express CYP3A4 in the presence of ^14^C-Gly. Cells then received 10 μM radicicol (an Hsp90 inhibitor) 1 h prior to the addition of 0 or 5 μM NOC18. The cells were harvested at the indicated times, and supernatants were analyzed to determine CYP3A4 ^14^C-heme content (*A*) and activity (*B*) at 6 h. Data are the mean ± SD; n = 3 experiments. ∗∗∗*p* < 0.001, ∗∗*p* < 0.01, ns, not significant, one-way ANOVA. ^14^C-Gly, ^14^C-glycine; CYP, cytochrome P450; GlyA-CHO, glycine auxotroph Chinese hamster ovary cell; Hsp90, heat shock protein 90; NO, nitric oxide; NOC18, 2,2′-(hydroxynitrosohydrazino)bis-ethanolamine; UT, untransfected cell samples.

We next tested if radicicol, which blocks Hsp90 function by inhibiting its ATPase activity (30) and blocks heme insertion into CYP3A4 and 2D6 during their normal maturation (15), would also block the NOC18-induced increase in CYP3A4 heme content and activity in the GlyA-CHO cells. Figure 7, *A* and *B* shows that radicicol addition completely prevented 5 μM NOC18 from causing a time-dependent increase in the ^14^C-heme content and activity of CYP3A4 that otherwise occurred over the 6 h exposure period. Western analyses showed that radicicol did not change the expression levels of CYP3A4 under any circumstance (Fig. S9). Thus, the NO-stimulated heme allocation into apo-CYP2D6 and 3A4 required the cell's Hsp90 ATPase activity to be functional.

## Discussion

CYP activities are NO sensitive, and a loss in their activities during infection and inflammation has been tied to an increase in host NO biosynthesis (31, 32, 33). NO can negatively impact CYP activities in different ways, including by binding to the CYP heme iron, causing CYP protein modifications like Tyr nitration and Cys nitrosation, or decreasing CYP gene expression or CYP protein lifetime (32). Our study reveals an additional way that NO can positively or negatively regulate CYP2D6 and 3A4 activities in cells, namely by causing their heme contents to increase or decrease. Low NO concentrations stimulated cells to allocate heme into their apo-CYP2D6 and apo-CYP3A4 populations, which increased the level of their heme-replete forms in the cells by twofold to threefold and led to corresponding increases in their catalytic activities. In cells exposed to NO at concentrations above this lower range, its ability to promote the cell heme allocation was gradually lost and at the higher concentrations ultimately caused no change and even caused a loss of heme from the CYPs and a corresponding drop in their activities. We observed similar effects for NO when it was released by NOC18 or was generated naturally by immune-activated macrophage cells in a coculture system. Thus, our work reveals that NO can both positively or negatively impact cellular CYP activities by causing an increase or decrease in their heme contents, which in turn depends on the NO concentration experienced by the cells.

In our experiments, we used GlyA-CHO and HepG2 cells grown under standard culture conditions, which thus contained their “normal” levels of heme. In this circumstance, the low-level NO exposure increased CYP2D6 and 3A4 heme content by twofold to threefold without changing their protein expression levels. This implies that 50% to 65% of the total CYP2D6 and 3A4 proteins expressed in either cell type were present in heme-free form prior to the NOC18 exposure. This matches with our previous finding that a similar percentage of heme-free CYP2D6 and 3A4 exists when they are expressed in the human embryonic kidney 293T cell line (15). It is also consistent with reports that CYPs expressed naturally in the mammalian brain and other organs exist significantly and even predominantly in their heme-free forms, with the extents of their heme deficiency varying depending on the tissue or mammal under study (4, 14, 34, 35). Because the heme-deficient CYPs in cells and tissues are inactive, this likely impacts their biological functions. Moreover, this creates an opportunity for biological levels of NO to upregulate or downregulate their heme contents and activities, as we demonstrated here for CYP2D6 and 3A4. Whether such NO-based regulation of CYP activities is operative in health and disease can now be further investigated.

It is remarkable how low an NO exposure could trigger cells to allocate heme into the apo-CYP enzymes. NOC18, which has the slowest release rate of any commercially available NO donor, caused cells to begin increasing their CYP heme contents and activities even at the lowest concentration used in our study (0.1 μM NOC18). At 5 μM NOC18, an optimal heme allocation occurred whose positive impact could already be seen after 2 h exposure. As noted in our previous report (26), based on an approximate 1000:1 relationship between the NOC18 concentration and the steady-state NO levels that are achieved in cultures (28), we surmise that steady-state NO concentrations of 100 pM to 5 nM NO stimulated cell heme allocation into apo-CYP2D6 and 3A4. These NO concentrations are similar to those needed to activate the NO receptor enzyme soluble guanylyl cyclase, are less than those needed to stimulate phosphorylation of extracellular signal–regulated kinase or Akt in cells, and are 20 to 80 times less than those needed to promote hypoxia inducible factor 1 alpha stabilization or P53 phosphorylation (28). Thus, promoting cell heme allocation may be among the most sensitive impacts of NO in biology.

For comparison, in our coculture experiments that included activated RAW264.7 macrophage cells, based on the measured nitrite that they generated within 6 h and assuming a nitrite:nitrate generation ratio of 3:2 for these cells (36), the range of total NO that they released over the 6 h coculture period was 8 to 100 μM. This is similar to the total NO released by the concentrations of NOC18 that we used, which we calculate to be 0.06 to 100 μM total NO released at 6 h, based on its measured half-life under our conditions (16 h) and on it releasing 2 mol NO per mole NOC18. This near equivalence in NO exposure helps to rationalize why cell heme allocation to the CYPs showed a similar hormetic pattern toward the NO generated by the range of NOC18 concentrations and to the NO generated by the range of macrophage cell numbers. Thus, the NO generated by chemical or cellular sources appeared to be functionally equivalent in how it affected cell heme allocation to the CYPs expressed within the GlyA-CHO cells.

Although the hormetic response that cell heme allocation displays toward the NO concentration may seem unusual, it actually mimics several other NO responses in biology where low NO concentrations have a beneficial effect, but as the concentrations increase, the effect disappears or reverses (37). Remarkably, the NOC18 concentration–response patterns of CYP3A4 and 2D6 were identical and remained so whether they were expressed in the GlyA-CHO or in the HEPG2 cell lines. Moreover, in our previous study of the heme proteins IDO1 and TDO, we observed highly similar NOC18 concentration–response patterns for their cell heme allocations in human embryonic kidney 293 cells, where 5 μM NOC18 was again optimal (26). Such high similarity among different heme proteins and cell types suggests that the NO concentration may influence cell heme allocation in ways that are independent of the cell type or the heme protein under study. This possibility can now be further investigated.

Regarding mechanisms, we found that the NO-driven cell heme allocation into CYP2D6 and 3A4 depended on GAPDH and its heme-binding ability and on the ATPase activity of Hsp90. The NO-driven heme allocation also caused Hsp90 to dissociate from CYP, thus generating a mature and catalytically functional enzyme. These findings imply that the low NO concentrations acted to stimulate the same cellular processes that naturally provide heme to these CYPs (15) and to other hemeproteins (27, 38, 39, 40, 41) during their normal maturation to functional forms.

NO exposure is known to increase the level of exchangeable heme in cells as detected by an intracellular heme sensor (42), and it also can speed heme transfer between purified proteins (43). Recently, the ability of NO to stimulate heme transfer from a GAPDH–heme complex to a client hemeprotein was tied to it, increasing the rate of heme dissociation from GAPDH (44). Whether these effects help to explain how low NO concentrations mobilized cell heme into the apo-CYP proteins remains to be investigated. It is interesting to note that the gain in CYP heme content upon low NO exposure did not reverse after it achieved its maximal level at 6 h. This implies that a range of low NO exposures exists where negative effects of NO on CYP heme allocations never manifest even with continued NO exposure. Whether this behavior relates to protective or repair processes operating within the cells deserves further study.

At the highest NO exposures (100 μM NOC18 or upon addition of the greatest number of activated macrophage cells), we saw that the heme-containing subpopulations of CYP2D6 and 3A4 began to lose their heme such that their heme levels ultimately diminished by approximately 50% to 75%. CYP heme loss was evident by the first 2 h of the high NOC18 exposure, suggesting there may have been no initial transient gains in their heme contents before the losses occurred. The experiments done in the presence of the protein synthesis inhibitor Chx only report on the behavior of existing CYP proteins, and because there was no change in the CYP expression levels or any cell toxic effect of the Chx, we can conclude that the losses in ^14^C-heme counts were not because of any CYP protein degradation. Thus, higher NO exposure caused the cells to “reallocate” heme out of their CYPs. This is consistent with our previous finding that higher NO exposures prevented cells from delivering heme into apo-CYP2D6 and 3A4 (25) and with an early report suggesting that NO may cause microsomal CYPs to lose heme (45) and the concept that CYP enzymes are in equilibrium with an exchangeable heme pool in cells (14). How higher NO exposures cause heme loss from the CYP proteins is unclear, but conceivably, it might involve a heme transfer back to GAPDH, as we recently found occurs for the IDO1 protein in cells (46) or alternatively, transfer to another entity like heme oxygenase-2. At this point, we surmise that the lower concentrations of NO promoted cell heme allocation into CYP2D6 and 3A4 by a mechanism that likely differs from how the higher NO concentrations caused these CYPs to lose their heme. The extent to which each mechanism operates in cells may presumably depend on what NO concentration they experience. It will now be important to investigate if NO-driven gains or losses in CYP heme content are common features among the larger protein family or among all the heme proteins whose heme deliveries have been found to be GAPDH dependent (27, 39, 40, 47).

**Figure 8:**
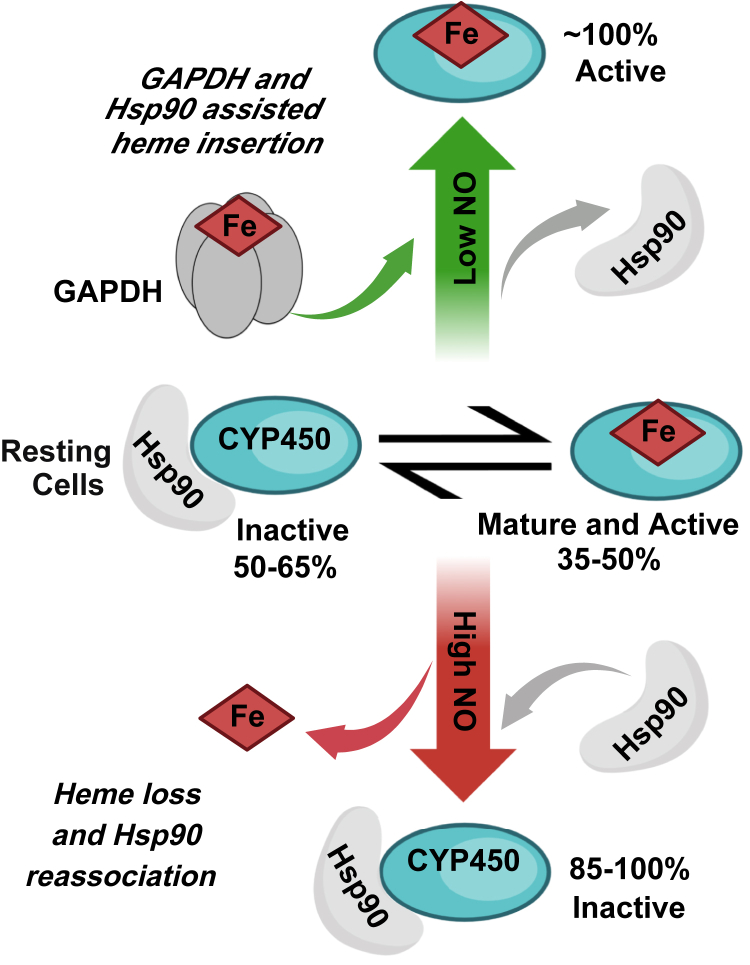
**Opposite effects of lowandhigh NO on cell heme allocation to CYP2D6 and 3A4, CYP-Hsp90 association, and catalytic activities.** In resting cells, the CYPs are present as a mix of their Hsp90-bound, heme-free inactive form and their Hsp90-free, heme-replete active form. Exposure to a low range of NO concentrations (approximately 1–10 nM) causes cells to allocate heme into the heme-free CYP population by NO stimulating heme delivery from a GAPDH–heme complex and heme insertion by Hsp90. This increases cell CYP activity. In contrast, exposure to higher NO concentrations (approximately 25–100 nM) causes the heme-replete CYP population to lose heme and to rebind Hsp90, and this decreases cell CYP activity. CYP, cytochrome P450; Hsp90, heat shock protein 90; NO, nitric oxide.

Together, our findings combined with previous studies improve our understanding of how NO can impact CYP3A4 and 2D6 in biological systems: These CYPs exist as a mixture of heme-free and heme-containing forms in cells and are thus subject to NO driving heme into or out of them depending on its concentration. This in turn upregulates or downregulates CYP3A4 and 2D6 activities in cells. Figure 8 illustrates the findings to date regarding cell heme allocation to CYP2D6 and 3A4 and the impact of NO.

The NO effects on CYP heme content may help to explain how their activities change in animals during chronic disease or inflammation (48, 49), which presents a confounding variable toward the development of effective and patient-specific pharmacologic therapies (50) and also impacts the biotransformation of polyunsaturated fatty acids and arachidonic acid into their active metabolites (51). Indeed, our demonstrating that a gradient of NO exposures as generated by different numbers of immune-stimulated macrophage cells could increase or decrease the heme content and activity of CYP3A4 expressed in neighboring cells may recapitulate what happens during inflammation-induced NO generation in mammals. Our findings also suggest how the reduction of tonic NO levels observed in old age or in some vascular and pulmonary diseases (52, 53, 54), and the excessive NO generation seen in some inflammatory diseases (32, 55, 56), could both be circumstances that reduce CYP heme contents and activities *in vivo*. Thus, NO upregulation and downregulation of CYP heme content has implications in biology, medicine, and pharmacology and could potentially be targeted to help preserve or modify CYP activities in health and disease.

## Experimental procedures

### Materials

^14^C-Gly (0.5 mCi) (15) was purchased from PerkinElmer, Inc. All other reagents used were purchased from Sigma, unless otherwise mentioned.

### CYP expression in GlyA-CHO cells and ^14^C-labeled heme synthesis

Ham's F12K or Kaighn's modified (37-500CUST, purchased from the Cleveland Clinic media core) containing 10% heme-depleted serum (57) was used to cultivate GlyA-CHO cells (glycine auxotrophic for growth and a gift from Dr P.J. Stover, Cornell University) in 10 cm tissue culture plates in a humidified incubator with 5% CO_2_ atmosphere at 37 °C. The cell cultures were given 400 μM of SA (D1415; Sigma) for 2 to 3 days to deplete their endogenous heme pool, and when they reached approximately 70% confluency, they were transfected with 10 μg of FLAG- and MYC-tagged CYP3A4 (RC210170; Origene) or CYP2D6 (RC223749; Origene) expression plasmids using Lipofectamine 2000 (11668019; Invitrogen). The protein expression was allowed to continue for 30 h in the presence or absence of SA. After that, the media in each plate were exchanged for 5 ml of F-12 medium without glycine (1242DMF-0823; Cell Culture Technologies LLC) with 10% heme-depleted serum and 2 μCi/ml of ^14^C-Gly and were further incubated for 24 to 30 h. At this point, the CYP proteins are produced and contain a baseline amount of ^14^C-heme. Cell cultures received 5 μg/ml of Chx (C7698; Sigma) to block further protein synthesis 6 to 8 h prior to initiating further experiments.

### CYP3A4 or 2D6 expression in HepG2 cells

HepG2 cells were cultured in Eagle's minimum essential medium (99BJ-500CUST) with 10% normal fetal bovine serum (Gibco). CYP3A4 expression was induced using 50 μM of rifampicin (R3501; Sigma) for 48 h. Since there was no noticeable CYP2D6 induction in response to rifampicin, the HEPG2 cells were transfected with 10 μg of FLAG- and MYC-tagged CYP2D6 plasmid following the protocol described previously for the GlyA-CHO cells.

### Cell harvest and supernatant preparation

Cells were harvested by removing the culture media from each plate and adding 150 μM of cold lysis buffer (40 mM 3-[4-(2-hydroxyethyl)piperazin-1-yl]propane-1-sulfonic acid [EPPS], 150 mM NaCl, 10% glycerol, pH 7.6) containing protease inhibitors (Roche). The cells were detached by scraping, and the resulting suspension underwent five freeze–thaw cycles, was centrifuged at 10,000*g* for 20 min at 4 C, and the supernatants were collected and kept at −80 °C until needed.

### RAW264.7 macrophage and GlyA-CHO cell coculture

We have followed the direct coculture system for our study. GlyA-CHO cells were grown and transfected with FLAG- and MYC-tagged human CYP3A4 expression plasmids in ^14^C-Gly-containing medium. Macrophage RAW264.7 cells (TIB-71; American Type Culture Collection) grown in separate 10 cm plates were activated with LPS (L2630; Sigma) and IFN-γ at 1 μg/ml for 6 h. After that, various concentrations (cell number counted in hemocytometer) of the activated (+LPS, +IFN-γ) and inactivated (−LPS, −IFN-γ) RAW264.7 cells were added to the plates with GlyA-CHO cells. The cells were kept in the incubator for 12 h. After 12 h, the media were collected, and nitrite accumulation in this medium was measured using the Griess reagent system (G2930; Promega). The plates were then harvested using cold lysis buffer (40 mM EPPS, 150 mM NaCl, 10% glycerol, pH 7.6) in a protocol mentioned previously. The cell supernatants were used in IP to measure ^14^C-heme counts and to determine FLAG-MYC-tagged human CYP3A4 activity and expressions by Western blot.

### Gene silencing and siRNA transfection

In 10-cm plates, GlyA-CHO cells were cultured and at 60% confluency transfected with commercially available siRNA against human GAPDH (D-001830-01-05; Dharmacon) and scrambled siRNAs (D-001810-10-05; Dharmacon) at a final concentration of 100 nM per plate using Lipofectamine-2000. After 48 h of growth, the siRNA-treated cells were transfected with plasmids expressing FLAG- and MYC-tagged human CYP3A4 proteins as previously mentioned.

### 3'-(4,5-Dimethylthiazol-2-yl)-2,5-diphenyl tetrazolium bromide cell viability assay

The cytotoxicity of Chx was assessed using the CyQUANT MTT Cell Viability assay (V13154; Invitrogen). The GlyA-CHO cells were seeded into a 96-well plate in 100 μl of cell culture medium and cultured with Chx at 5 μg/ml for different time intervals (0, 2, 4, 6, 12, and 24 h) in a CO_2_ incubator at 37 °C. 3′-(4,5-Dimethylthiazol-2-yl)-2,5-diphenyl tetrazolium bromide stock solution (5 mg/ml, 10 μl) was added to each well and incubated in the dark for 4 h at 37 °C. The solution was aspirated and replaced with 100 μl of dimethyl sulfoxide to dissolve the formazan, and the absorbance at 570 nm was obtained using a microplate reader. The viability of the cells was calculated based on the absorbance of control cell wells that did not receive Chx. Cell viability = (Absorbance of treated cells-blank absorbance)/(Mean absorbance of control cells-blank absorbance) × 100.

### Immunoprecipitation

GlyA-CHO cells were lysed using 40 mM EPPS, 150 mM NaCl, 10% glycerol, pH 7.6 and EDTA-free protease inhibitor cocktail (Roche). Protein concentration was measured using the Bradford method (500-0006; Bio-Rad). IPs were carried out using protein G Agarose beads (16-201; Millipore). The cell supernatant (1 mg protein) was combined with 3 μg of antibodies against MYC tag. The combination was then rotated at 4 °C for 1 h, followed by the addition of 70 μl of Protein G Agarose beads, and the mixture was rotated at 4 °C overnight, followed by centrifuging the beads at 700*g* and washing them three times with cold buffer (40 mM EPPS, 150 mM NaCl, 10% glycerol, pH 7.6). The washed beads were then either mixed with scintillation fluid (liquiscint, LS-121; National Diagnostics) for ^14^C radioactive counting, or they were boiled with Laemmli buffer, centrifuged, and the solution was loaded onto SDS-PAGE gels.

### Western blotting

Cells were lysed in cold buffer (40 mM EPPS, 150 mM NaCl, 10% glycerol, pH 7.6) containing protease inhibitors (Roche) and then by five freeze–thaw cycles, then centrifuged at 10,000*g* for 20 min, and the supernatants were collected. Their protein concentration was determined using the Bradford technique (500-0006; Bio-Rad). For each sample, 30 μg of protein was boiled in Laemmli buffer and resolved using 10% SDS-PAGE, and the proteins were transferred to polyvinylidene difluoride membranes (1620177; Bio-Rad). After 30 min of blocking the membranes with 5% nonfat blocking grade milk (1706404; Bio-rad), primary antibodies against MYC (Cell Signaling Technologies; 1:1000 dilution), GAPDH (Cell Signaling Technologies; 1:2500 dilution), iNOS (MAB9502; R&D Systems; 1:1000 dilution), and β-Actin (A5441; Sigma; 1:2500 dilution) were added. Horseradish peroxidase–conjugated secondary antibodies of either anti-mouse (170-6516; Bio-Rad; 1:5000 dilution) or anti-rabbit (NA9340; GE Healthcare; 1:5000 dilution) origin and enhanced chemiluminescence substrate (32106; Thermo Scientific) were used in chemiluminescence to detect the proteins. A ChemiDoc system from Bio-Rad and ImageJ (NIH) software were used to capture the images and quantify band intensities.

### NOC18 treatment of cells

NOC18 (N379-12; Dojindo Molecular Technologies) was freshly dissolved in cell culture grade sterile PBS at a stock concentration of 10 mM; then this was diluted into phenol red–free cell culture media containing 10% serum to give concentrations of 2 mM or less, and these solutions were then added to cells in culture to generate the final concentrations.

### Radicicol treatment of cells

GlyA-CHO cells were grown and transfected with the FLAG- and MYC-tagged human CYP3A4 expression plasmid in ^14^C-Gly-containing medium. They were then incubated for 1 h with or without radicicol at 10 μM (R2146; Sigma) and then given 0 or 5 μM NOC18. Cells remained in the presence of radicicol for the duration of the experiment.

### Cyp3A4 and CYP2D6 activity assays

The CYP3A4 and CYP2D6 activities in cell supernatants were measured using fluorometric assay kits from Abcam (ab211076 and ab211078). The kit protocol was followed, utilizing around 50 μg of supernatant protein in a final volume of 70 μl in each well. The culture plates were scanned using the POLARstar microplate reader in the kinetics mode, and fluorescent change was recorded for 30 to 45 min at 37 °C using excitation/emission wavelengths of 535/587 nm for CYP3A4 and for 60 min at 37 °C using excitation/emission wavelengths of 390/468 nm for CYP2D6. The activities were determined based on the slopes and a standard plot using resorufin for CYP3A4 and AHMC for CYP2D6 and are expressed as picomole product/minute/microgram of protein.

### Statistical analyses

All studies were performed in three independent trials, each with three replicates. The data are shown as the average of the three trial values ± SD. The statistical test used to determine significance (*p* values) was one-way ANOVA in GraphPad Prism (version 9; GraphPad Software, Inc). A *p* value of less than 0.05 was considered statistically significant.

## Data availability

All data are contained within the article or are available from the authors upon request.

## Supporting information

This article contains supporting information.

## Conflict of interest

The authors declare that they have no conflicts of interest with the contents of this article.
