# Supplementary material for: Nitric oxide regulates cytochrome P450 2D6 and 3A4 activity via concentration-dependent modulation of heme loading
Source: J Biol Chem. 2025 Sep 27;301(11):110772. doi: 10.1016/j.jbc.2025.110772 (PMC12605048; doi:10.1016/j.jbc.2025.110772)
Supplement: Supporting information [file mmc1.pdf]

Nitric Oxide Regulates Cytochrome P450 2D6 and 3A4 Activity via Concentration-Dependent Modulation of Heme Loading

Priya Das Sinha, Sidra Islam, Pranjal Biswas and Dennis J. Stuehr[1*]

**Figure Number**	**Page Number**
Fig. S1	S2
Fig. S2	S3
Fig. S3	S4
Fig. S4	S5
Fig. S5	S6
Fig. S6	S7
Fig. S7	S8
Fig. S8	S9
Fig. S9	S10

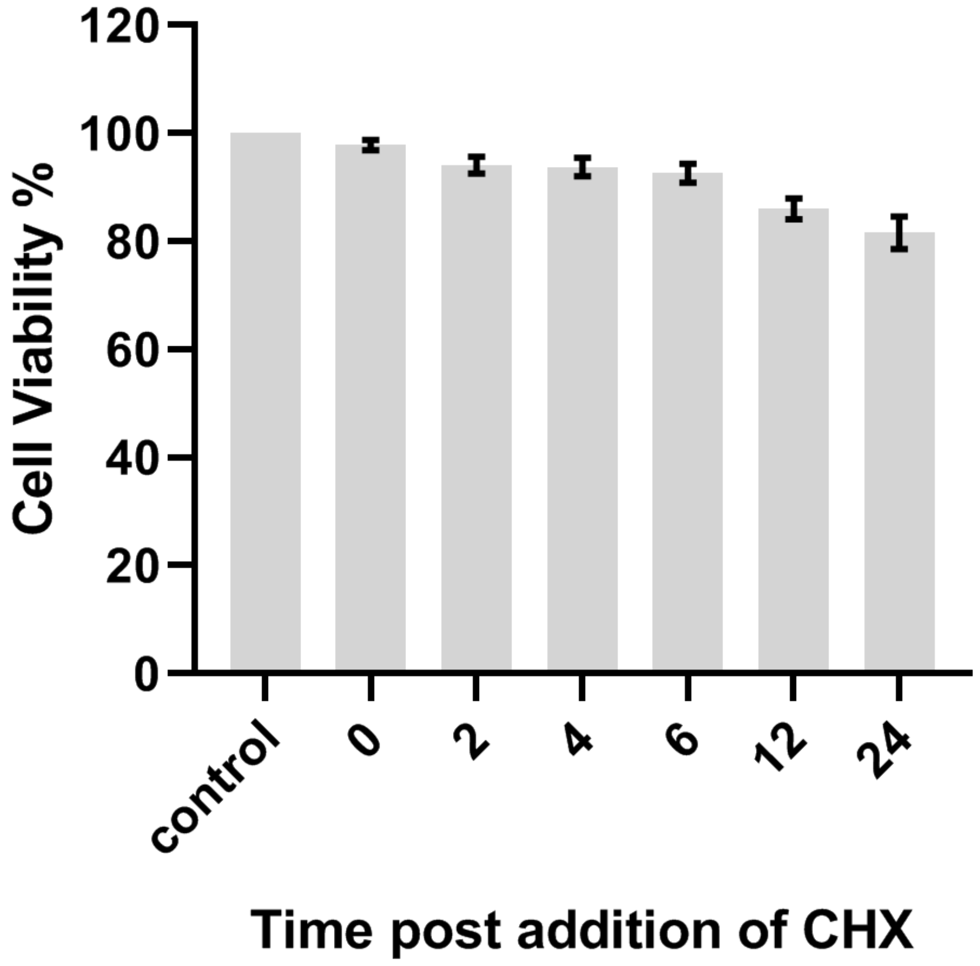

Fig. S1. Cycloheximide at our working concentration does not exert a cytotoxic effect. GlyA-CHO cells expressing CYPs in 96 well plate were given Chx to cease protein expression at a concentration of 5µg/ml for various time intervals (2,4,6,12,24 h) to demonstrate its effect on cell survival. The MTT cell viability assay was conducted and absorbance measurement at 570nm were made using a microplate reader. Data represents the mean +/- SD of three independent experiments.

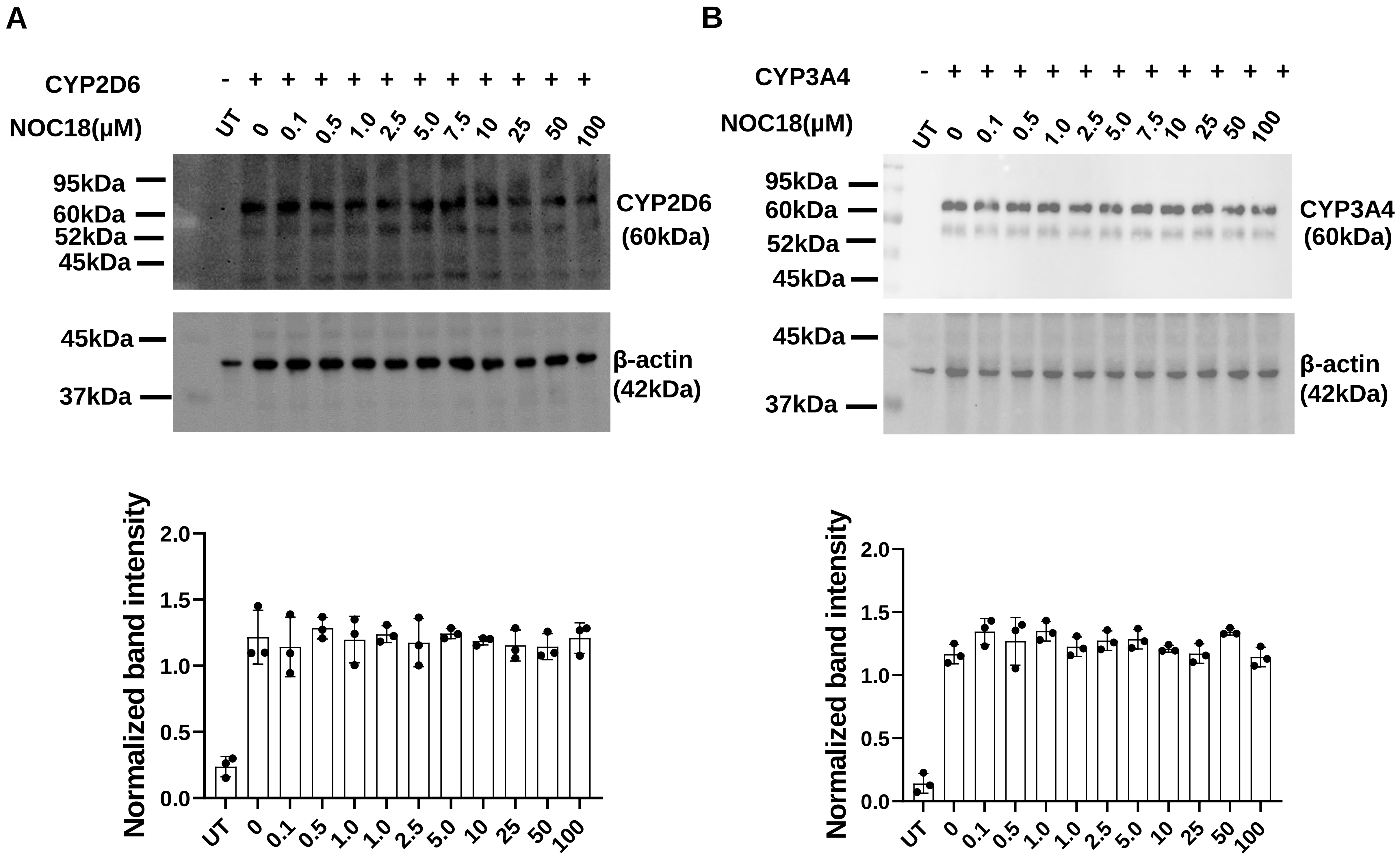

Fig. S2. CYP2D6 (A) and CYP3A4 (B) protein expression levels in GlyA-CHO cells after they underwent incubation with the indicated range of NOC18 concentrations. Representative Western blots are shown. Data coincides with the sample data in main Fig.1 and represent the mean +/- SD of three independent experiments.

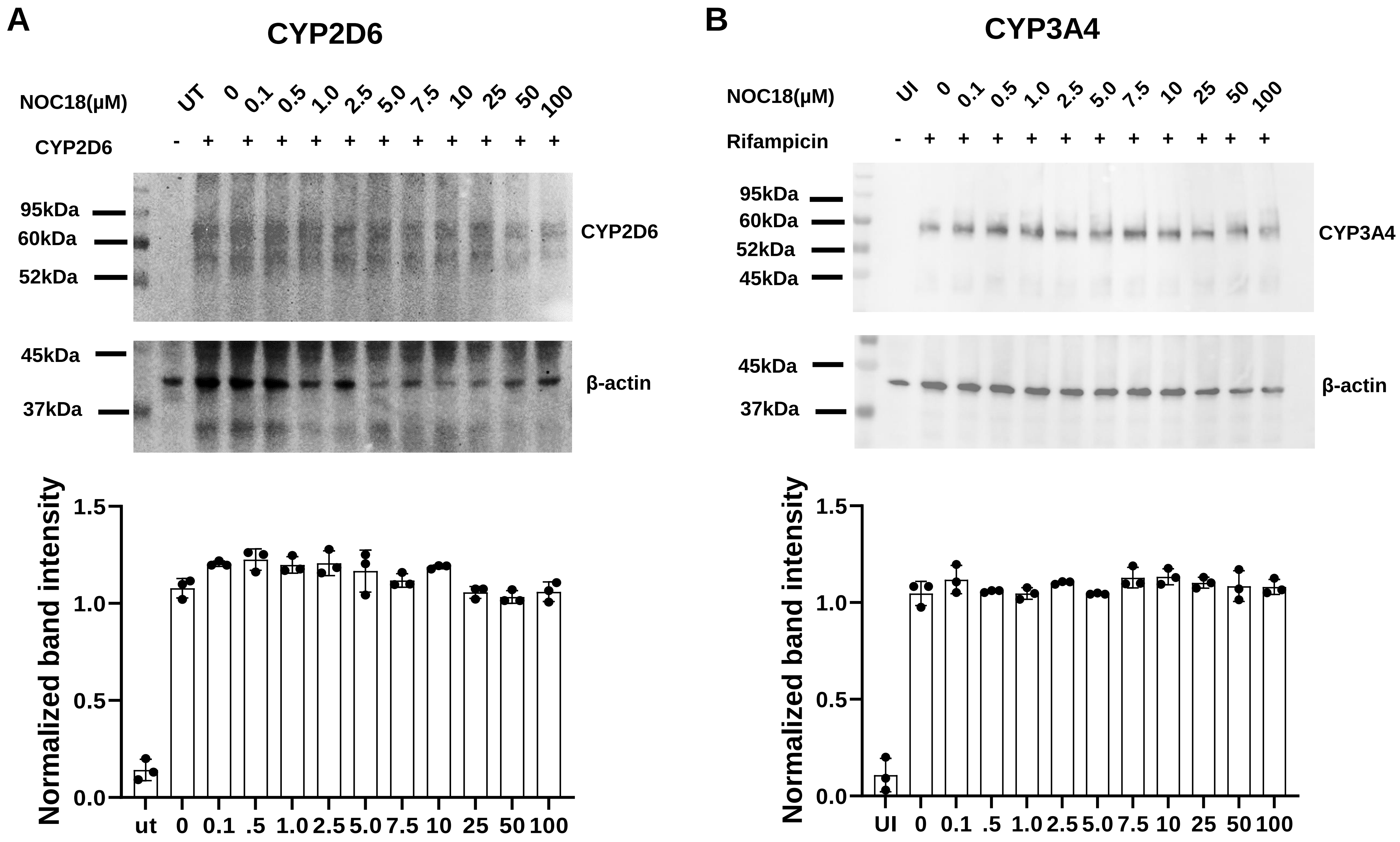

Fig. S3. CYP2D6 (A) and CYP3A4 (B) protein expression levels in HepG2 cells after they underwent transfection with plasmid or induction by rifampicin respectively followed by incubation with the indicated range of NOC18 concentrations. Representative Western blots are shown. Data coincides with the sample data shown in main Fig.2 and represents the mean +/- SD of three independent experiments.

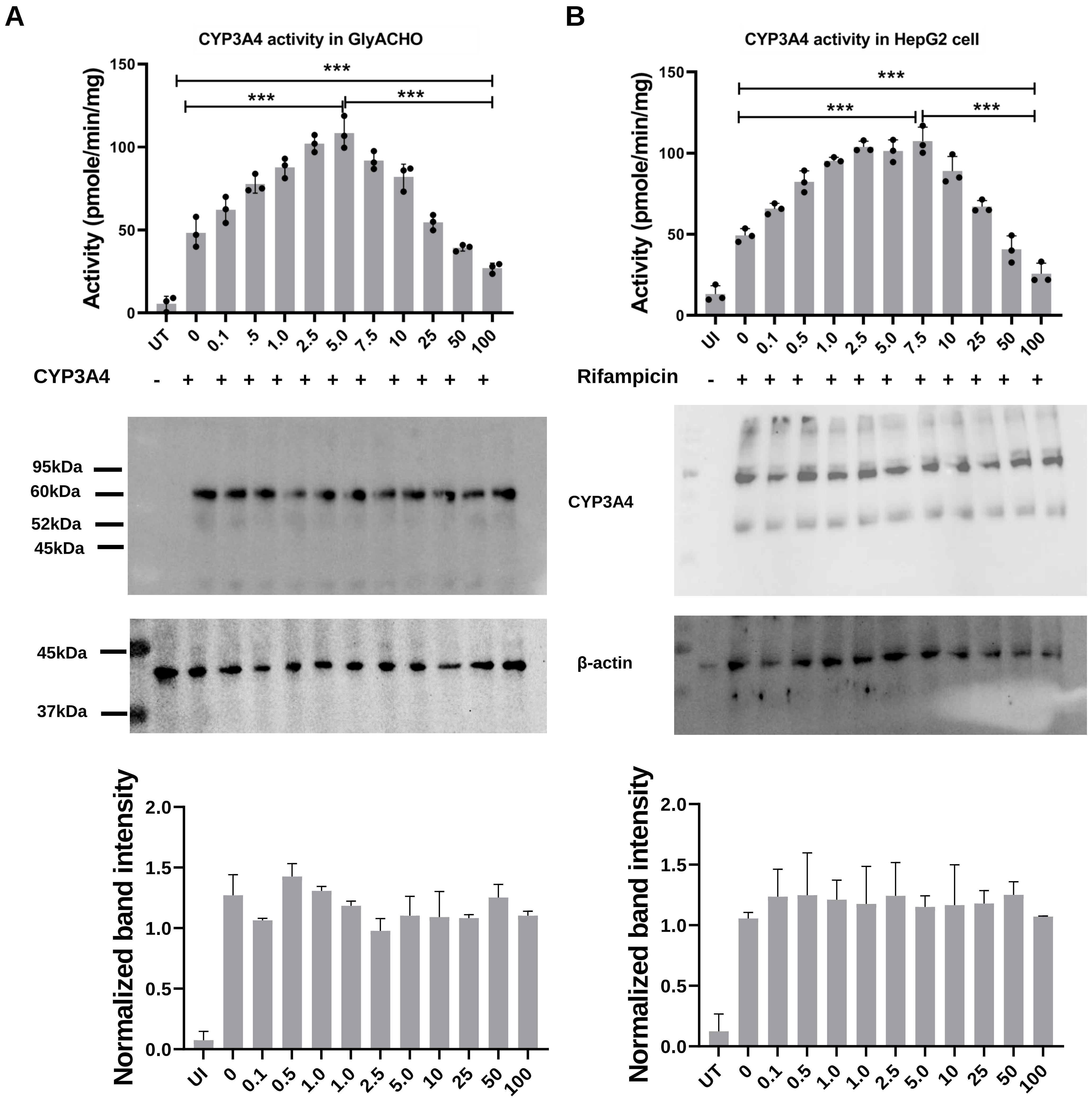

Fig. S4. CYP3A4 activity and protein expression in GlyACHO and HepG2 cells in replica experiments using cell cultures that did not receive Chx. Cells were exposed to the indicated NOC18 concentrations for 6 h and then lysed. Supernatant activities and CYP expression levels were compared, representative Western blots are shown. Data represents the mean +/- SD of three independent experiments.

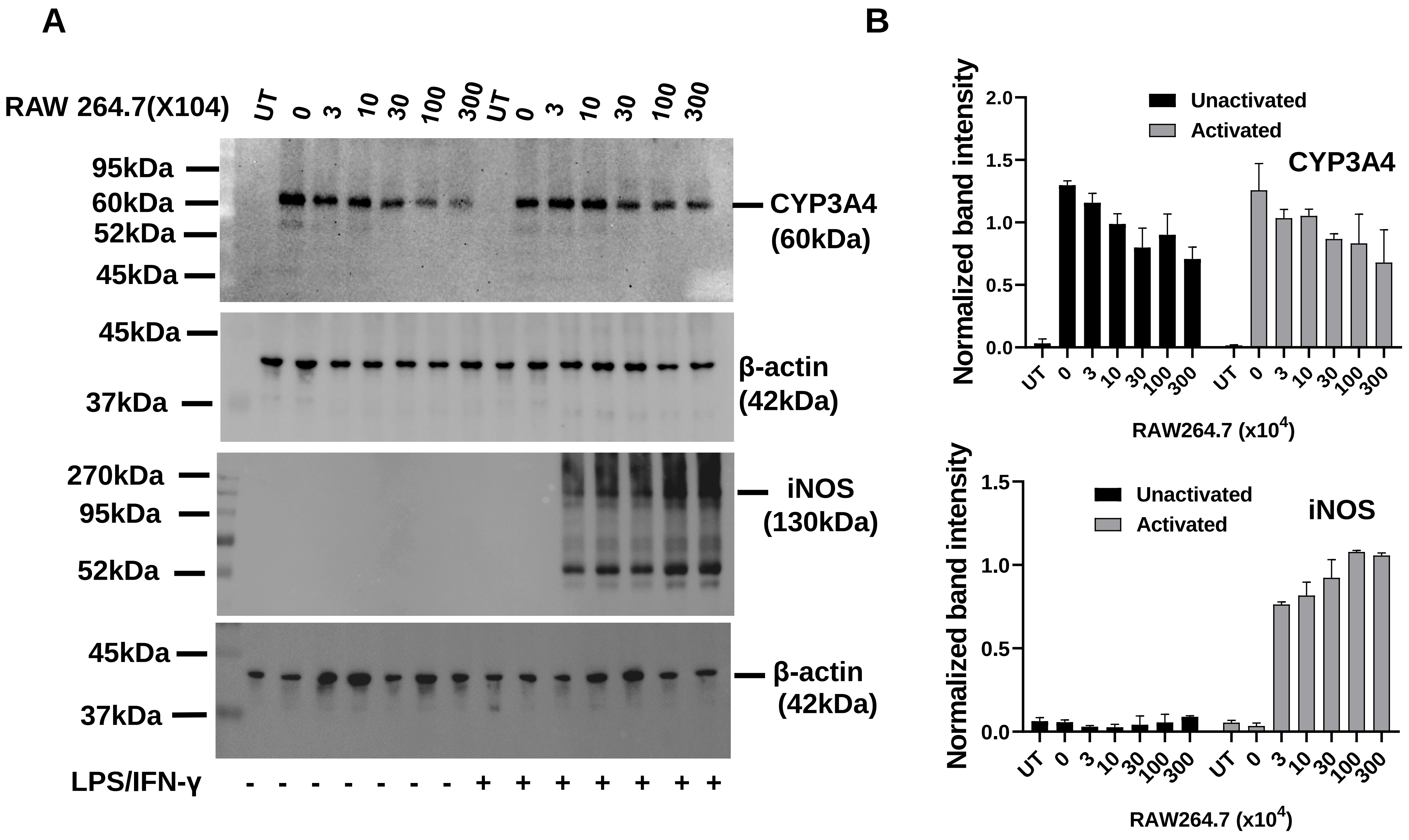

Fig. S5. **Expression levels of CYP3A4 and iNOS in co-cultures containing GlyA-CHO cells and the indicated number of either non- or LPS/IFN-gamma-activated RAW264.7 cells.** GlyA-CHO cells were transfected to express CYP3A4 prior to co-culture. (A) Representative Western blots. (B) Corresponding normalized band intensities. UT, untransfected cells. Data are the mean ± SD; n = 3 independent experiments and correspond with the sample data shown in main Fig. 3.

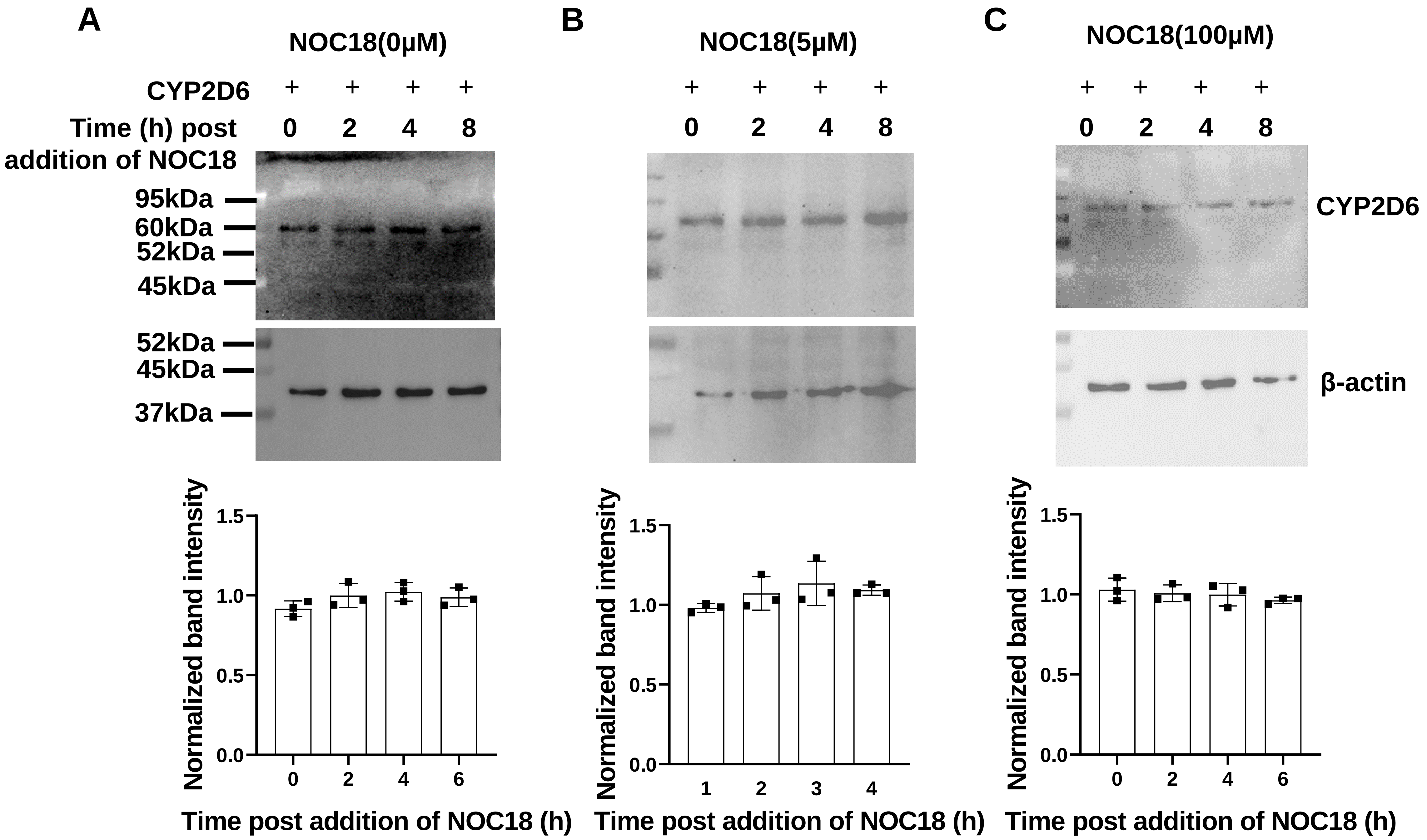

Fig. S6. Expression levels of CYP2D6 in GlyA-CHO cells during NOC18 treatment. GlyA-CHO cells were transfected to express CYP2D6, protein synthesis was then blocked with Chx, and NOC18 was added at concentrations of 0 (A), 5 (B), and 100 (C) μM, followed by harvesting and analysis of the cell supernatants at the designated time points. Representative Western blots are shown. Data are the mean ± SD; n = 3 experiments and correspond to data shown in main Fig. 4A and B.

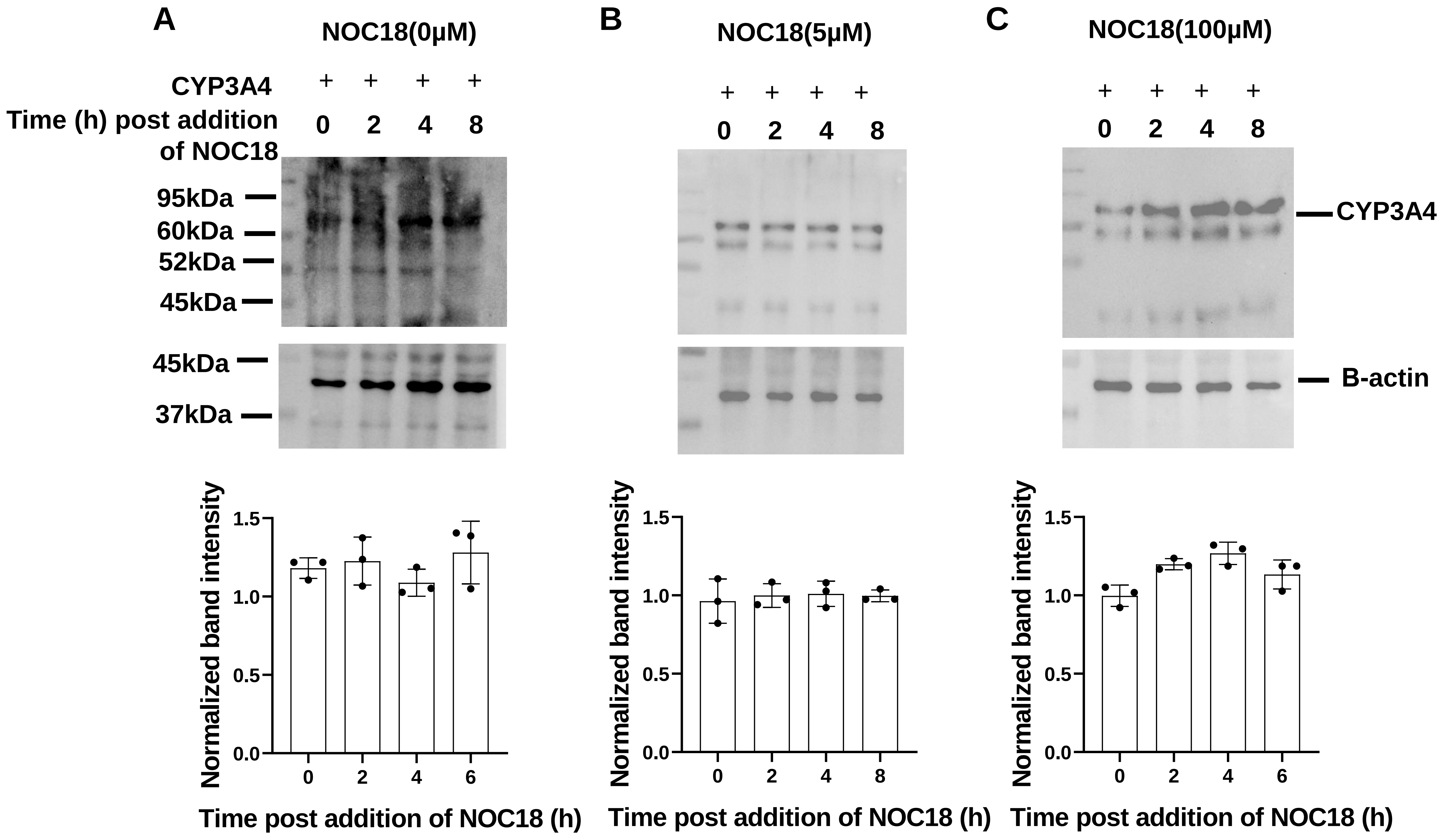

Fig. S7. Expression levels of CYP3A4 in GlyA-CHO cells during NOC18 treatment. GlyA-CHO cells were transfected to express CYP3A4, protein synthesis was then blocked with Chx, and NOC18 was added at concentrations of 0 (A), 5 (B) and 100 (C) μM, followed by harvesting and analysis of the cell supernatants at the designated time points. Representative Western blots are shown. Data are the mean ± SD; n = 3 experiments and correspond to sample data shown in main Fig. 4C and D.

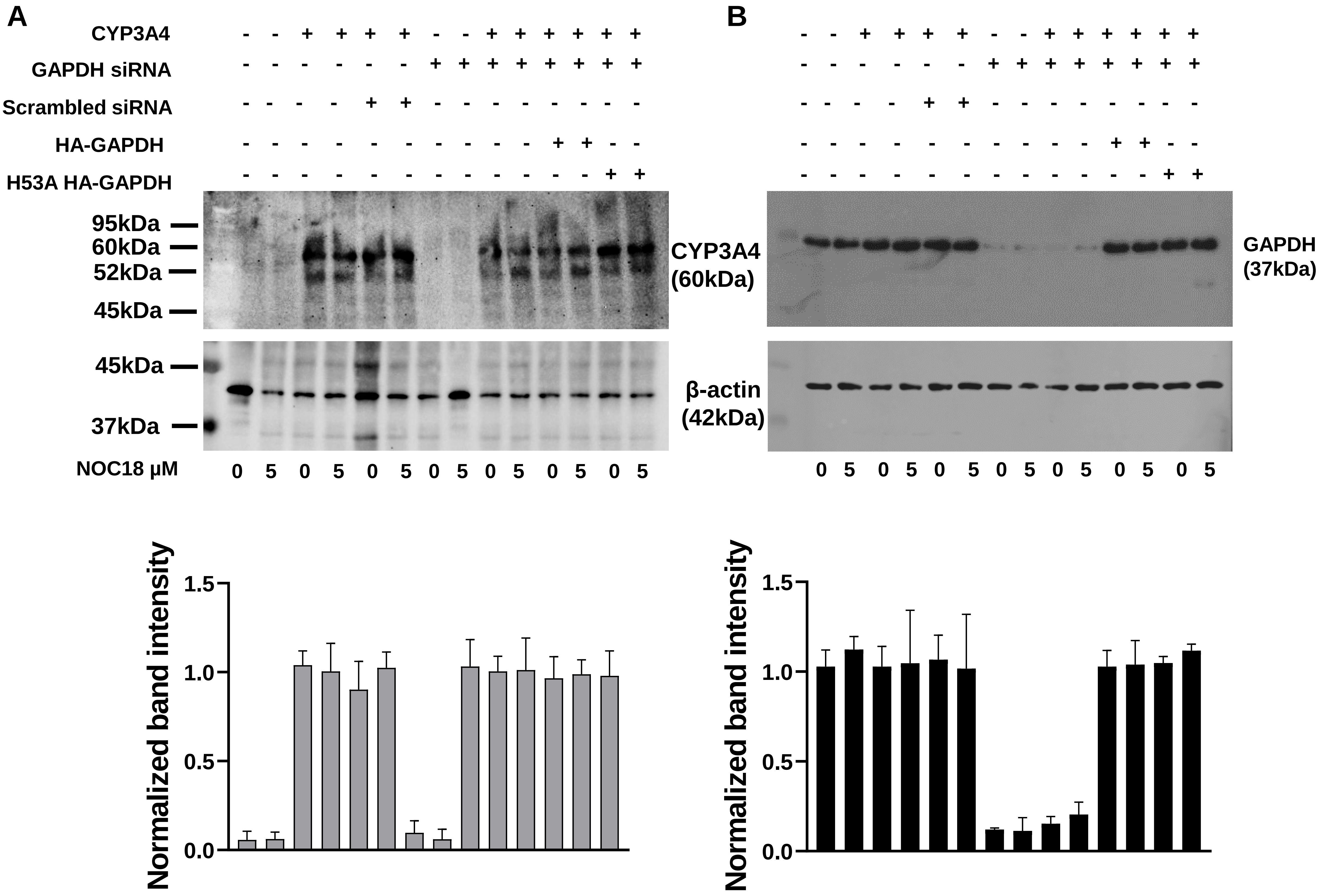

Fig. S8. CYP3A4 and GAPDH expression levels in GlyA-CHO cells that underwent the indicated treatments. GlyA-CHO cells that had been subjected to siRNA knockdown of GAPDH expression or treated with scrambled siRNA were subsequently transfected to express CYP3A4 either alone or along with siRNA-resistant versions of wild type HA-GAPDH or the heme-binding defective HA-GAPDH-H53A variant. Cells were subsequently treated with 0 or 5 μM NOC18, and after 6h the cell supernatants were analyzed for CYP3A4 (A) or GAPDH (B) expression. Representative Western blots are shown. Data are the mean ± SD; n = 3 experiments and correspond to sample data shown in main Fig. 5.

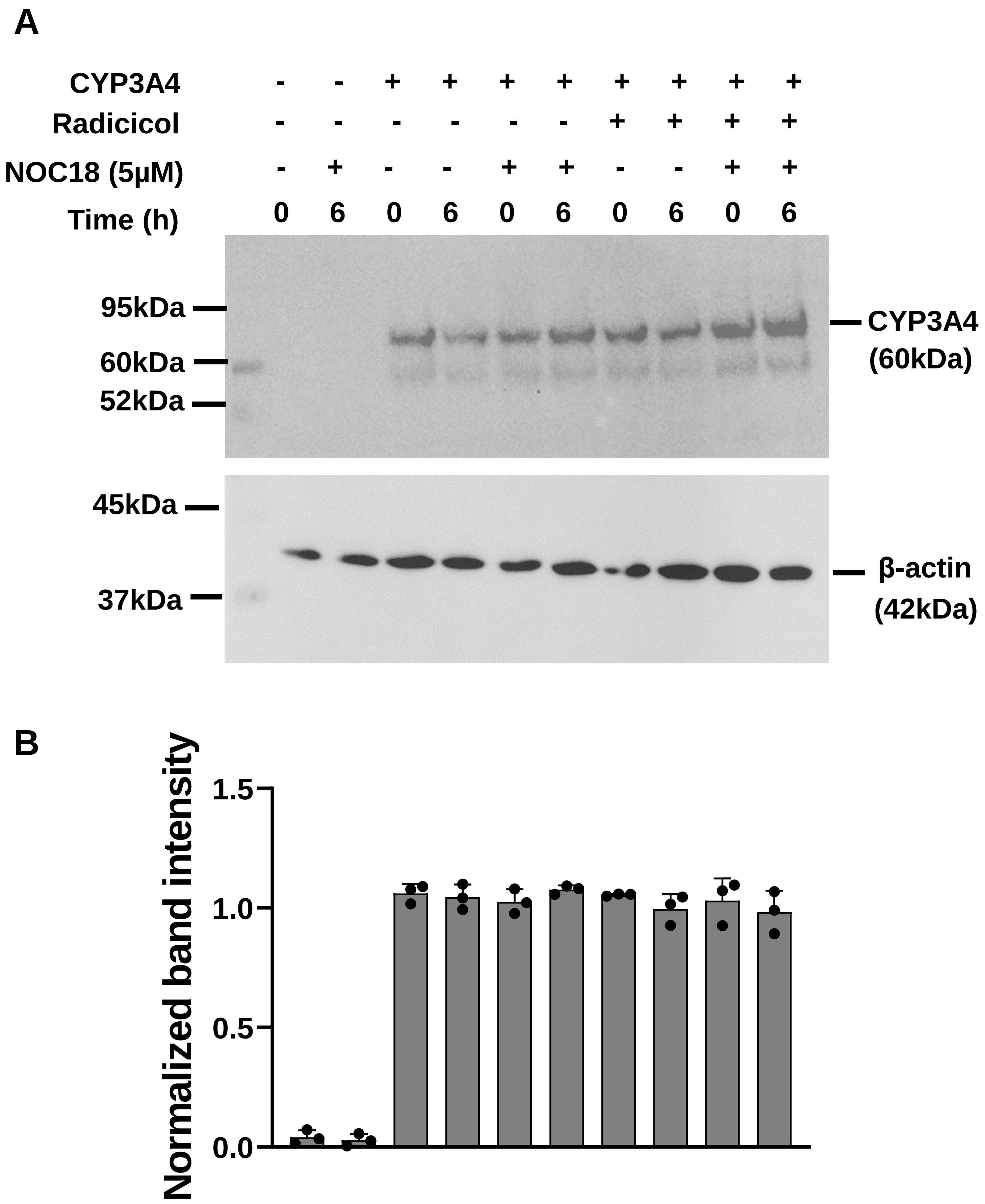

Fig. S9. CYP3A4 expression levels in GlyA-CHO cells after Radicicol pre-treatment followed by incubation with or without NOC18. GlyA-CHO cells underwent transfection to express CYP3A4, then received 10 µM radicicol 1h prior to receiving 0 or 5 µM NOC18. The cells were harvested after 0 or 6 h and supernatants analyzed to determine CYP3A4 expression levels. (A) Representative Western blot. (B) Normalized CYP3A4 band intensities. Data are the mean ± SD; n = 3 experiments and correspond to sample data shown in main Fig. 7.
